# Progression of experimental chronic Aleutian mink disease virus infection

**DOI:** 10.1186/s13028-016-0214-7

**Published:** 2016-06-01

**Authors:** Trine Hammer Jensen, Mariann Chriél, Mette Sif Hansen

**Affiliations:** 1 National Veterinary Institute, Technical University of Denmark, Bülowsvej 27, 1870 Frederiksberg C, Denmark; 2 Department of Chemistry and Bioscience, Aalborg University/Aalborg Zoo, Frederik Bajers Vej 7H, 9100 Aalborg, Denmark; 3 Aalborg Zoo, Mølleparkvej 63, 9000 Aalborg, Denmark

**Keywords:** Aleutian mink disease virus, AMDV, Mink, Chronic experimental infection, Histopathology

## Abstract

**Background:**

Aleutian mink disease virus (AMDV) is found world-wide and has a major impact on mink health and welfare by decreasing reproduction and fur quality. In the majority of mink, the infection is subclinical and the diagnosis must be confirmed by serology or polymerase chain reaction (PCR). Increased knowledge based on a systematically description of clinical signs, pathology and histopathology might be a tool to reduce the risk of infection from subclinically infected mink to AMDV free herds. The aim of this study was to give a histopathological description of the progression of a chronic experimental infection with a currently circulating Danish strain of AMDV, Saeby/DEN/799.1/05. These results were compared with the pathogenesis of previously published AMDV stains.

**Results:**

This experimental AMDV infection resulted in only decreased appetite and soft or discolored feces, primarily within the first 8 weeks after AMDV inoculation. Gross pathology revealed few and inconsistent findings mainly associated with the liver, spleen and kidneys. The majority of the AMDV inoculated wild type mink (n = 41) developed various histopathological changes consistent with AMDV infection in one or more organs: infiltrations of mononuclear cells in liver, kidney and brain, reduced density of lymphocytes and increased numbers of plasma cells in lymph nodes and spleen. Natural infection, as occurred in the sentinel sapphire mink (four of six mink), progressed similar to the experimentally inoculated mink.

**Conclusions:**

Experimental AMDV inoculation mainly resulted in subclinical infection with unspecific clinical signs and gross pathology, and more consistent histopathology appearing at any time after AMDV inoculation during the 24 weeks of observation. Thus, the observed histopathology substantiates AMDV infection and no correlation to time of inoculation was found. This confirms that diagnosing AMDV infection requires serology and/or PCR and the Saeby/DEN/799.1/05 AMDV strain results in histopathology consistent with other AMDV strains.

## Background

Aleutian mink disease virus (AMDV) is a parvovirus infecting mink (*Neovison vison*) world-wide [1–4]. AMDV infection is characterized by plasmacytosis, hypergammaglobulinaemia and immune complex formation [5]. The outcome of AMDV infection depends on host factors such as genotype and the age of the mink, viral load and the virus strain [6–8]. In juvenile mink, AMDV infection may cause acute pneumonia with high mortality [9–11]. In adult mink, one or more of the following conditions can occur as acute though mainly chronic lesions: glomerulonephritis [5, 12], arteritis [13], hepatitis [14] and meningoencephalitis [15, 16]. In naïve mink, acute pneumonia [9–11] and facial swelling (unpublished observations, the authors) can be the predominant clinical sign of all age groups. Classical chronic symptoms of AMDV infection is single white hairs in the fur (“sprinklers”) [17], reduced reproductive performance including reduced litter size and abortions [10]. Histologically, evidence of AMDV infection is interstitial pneumonia with alveolar hyaline membranes in mink kits [10] or plasma cell infiltrations in various organs in adult mink [5, 11]. However, polymerase chain reaction (PCR) or serology must be applied to confirm the diagnosis [1, 18–20].

Most AMDV infected farmed mink develop subclinical infections, but this is not well documented in recent literature [21]. Subclinically infected mink poses the main risk of transmitting AMDV to uninfected mink farms. Previous experimental studies on AMDV histopathology involve older strains of AMDV and focus on specific organs [22, 23] and not on the disease progression in the whole organism. The aim of the present study was to describe lesions and disease progression induced by a currently circulating Danish AMDV strain, Saeby/DEN/799.1/05. These results would be of practical diagnostic value and would in comparison with previously described AMDV strains reveal if potential consistent clinical signs or lesions could indicate the time of infection. During this chronic experimental AMDV infection of Danish farmed mink, the mink were monitored daily for clinical signs and evaluated for gross pathology and histopathology 2, 4, 8, 16 and 24 weeks after inoculation.

## Methods

### Mink

Mink (*Neovison vison*) were purchased from two farms on Zealand (Denmark), an island that at the time of purchase and during this study had been free of AMDV for more than a decade. The mink (42 wild type mink and 6 sapphire mink) were tested negative for antibodies against AMDV by counter-current immunoelectrophoresis (CIEP) before they were moved to the experimental facility at the National Veterinary Institute (NVI), Technical University of Denmark. The mink were all females because they were easier to handle in terms of size and temper. Twelve one-year-old wild type mink were used for a short term infection experiment. Half of the mink (n = 15 wild type mink, n = 3 sapphire mink) used in the chronic infection experiment were five-months-old and the other half of the mink (n = 15 wild type mink, n = 3 sapphire mink) were 15-months-old. The farms had no records of previous AMDV, mink enteritis virus or other serious infectious diseases and no records of any vaccinations. The care and use of the animals was in accordance with the guidelines and approved by The Animal Experiments Inspectorate (approval number 2009/561-1755).

The 48 mink were housed indoor in four isolation rooms designed for animal experiments with 12 mink cages in one row in each room. Each mink was housed individually in standard mink wire mesh cages with a wooden nest box with straw. A standard commercial mink diet from the local mink feed producer were given to the mink daily. Upon arrival at the animal experimental facility, all mink were clinically assessed and the mink had 2 days for acclimatization before AMDV inoculation at day 0. Mink exhibiting clinical signs according to clinical endpoints (weight loss, anorexia, compromised welfare, or severe gastrointestinal, respiratory or urinary symptoms) at any time during the experiment were euthanized. One wild type mink was euthanized before the AMDV inoculation for health reasons and thus not included in the data analysis. Two sapphire mink were included in each group as sentinels. Sapphire mink carry the Aleutian gene making them more susceptible to Aleutian disease (AD) [7, 8].

### Experimental infections with AMDV

A short term experiment of 1 month with 12 one-year-old wild type female mink was performed to determine the lowest dilution of the AMDV organ homogenate for induction of chronic infection. The mink were divided into four groups of three mink that were anesthetized by intramuscular injection with ketamine (10–15 mg/kg) and xylazine (0.5–1 mg/kg), followed by intraperitoneal injection of 1 ml of the AMDV organ homogenate undiluted, diluted in PBS with 4 % penicillin/streptomycin 1:100, 1:1000 or 1:10,000. The homogenate consisted of spleens from farmed mink naturally infected in 2009 with the currently most prevalent Danish AMDV strain Saeby/DEN/799.1/05 [4] as described by Jensen et al. [24]. The mink were observed daily and clinically scored as described below. Blood samples, fecal samples and oro-nasal swabs were collected weekly.

**Fig. 1:**
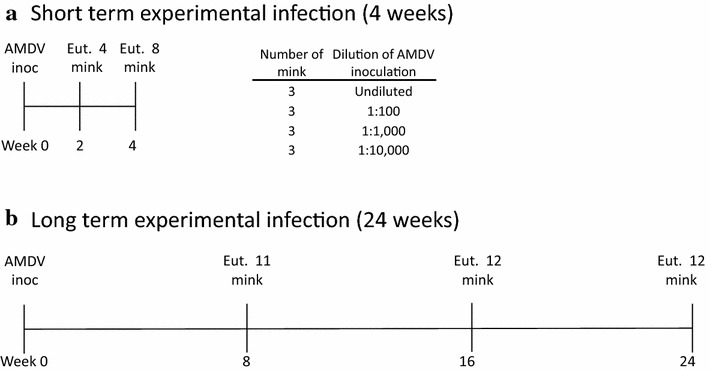
Mink inoculated with Aleutian mink disease virus (AMDV) and euthanized 2 and 4 weeks later (**a**) and 8, 16 and 24 weeks later (**b**). The mink in experiment A were given different dilutions of AMDV as shown in the table. One mink from each dilution group was euthanized after 2 weeks and the rest after 4 weeks. The mink in experiment B were inoculated with a 1:10,000 dilution of the AMDV homogenate. *Inoc* inoculated, *Eut* euthanized

Two weeks after AMDV inoculation one mink from each of the four groups were euthanized by cardiac injection of pentobarbital preceded by ketamine/xylazine anesthesia. The remaining eight mink were euthanized 4 weeks after AMDV inoculation (Fig. 1). All mink were necropsied and organs were examined macroscopically, sampled and stored at −80 °C. One mink delivered two mink kits 12 days after AMDV inoculation from which oro-nasal swabs and organs were sampled.

**Fig. 2:**
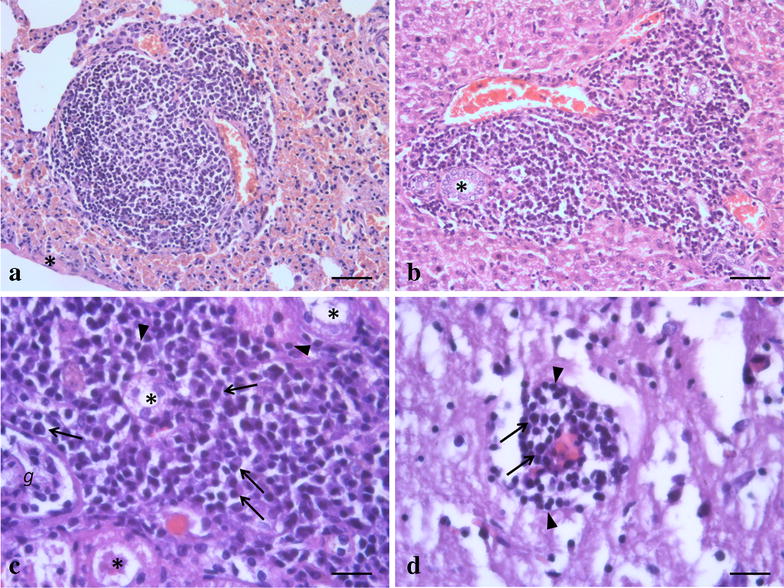
Histopathological findings in wild type mink experimentally inoculated with AMDV. **a** Massive perivascular infiltration of mononuclear cells in the lung 8 weeks after AMDV inoculation. Pleura (*asterisk*). *HE* Hematoxylin and eosin. *Scale bar* 50 µm. **b** Massive infiltration of mononuclear cells in a portal triad of the liver 4 weeks after AMDV inoculation. Bile duct (*asterisk*). HE, *scale bar* 50 µm. **c** Massive interstitial infiltration of mononuclear cells in the cortex of the kidney 16 weeks after AMDV inoculation. Plasma cells (*arrow*), lymphocytes (*arrowhead*), kidney tubule (*asterisk*), glomerulus (*g*). HE, *scale bar* 20 µm. **d** Moderate non-suppurative perivascular cuffing in white matter of cerebrum 16 weeks after AMDV inoculation. Plasma cells (*arrow*), lymphocytes (*arrowhead*). HE, *scale bar* 20 µm

A long term chronic infection experiment of  6 months was initiated by intraperitoneal inoculation of 29 wild type female mink, and six sapphire female mink were used as sentinels as described by Jensen et al. [24]. The 35 mink were divided in three groups with 11, 12 and 12 mink in each and euthanized after 8, 16 and 24 weeks respectively (Fig. 2). Details of this experiment are described by Jensen et al. [24]. In short, during the experiment blood samples were taken weekly to test for AMDV antibodies by CIEP and AMDV DNA by PCR for the first 8 weeks and then every second week till the end of the experiment at 24 weeks after AMDV inoculation. Oro-nasal swabs and feces were collected at the same days and tested for AMDV DNA. At the end of the experiment the mink were euthanised, necropsied and all organs were examined for AMDV DNA by PCR.

### Scoring of clinical signs

Every day each mink were clinically assessed and scored according to: (a) general condition, (b) respiration, (c) appetite, (d) feces. A score of 0–3 was given for each category depending on the clinical status with 0 describing the normal general condition with: (a) curiosity and free movements, (b) normal respiration with a moist nose, (c) eating all feed at once, and (d) formed dark normal feces. The score 1 was given for slight symptoms such as: (a) slightly depressed, slow and reduced movements, (b) labored respiration, (c) not eating all feed at once, (d) scarce dry or soft feces. The score 2 was given for moderate symptoms such as: (a) depression, only moves when forced, (b) decreased appetite, (c) labored respiration with sneezing and/or nasal discharge, and (d) either very scarce, dry feces or mild to moderate diarrhea. The score 3 was given for severe clinical signs involving (a) apathy/lethargy, (b) heavy respiratory sounds with nasal discharge and coughing, (c) anorexia, and (d) no feces or severe diarrhea. Finally, the content of blood in the urine was scored visually with 0 if unapparent or 3 if hematuria.

### Histopathology, detection of AMDV DNA and antibodies

At necropsy samples of lung, liver, spleen, duodenum, jejunum, mesenteric lymph node, kidney and brain (cerebrum and cerebellum) were fixed by immersion in 10 % neutral buffered formalin, processed by routine histology methods, embedded in paraffin wax and cut in 3–5 μm sections. Not all organs were collected from every mink (Table 4). The sections were mounted on conventional glass slides and stained with hematoxylin and eosin for histopathological examination. The tissue sections were evaluated blinded and systematically for the following AMDV associated lesions [23]: perivascular infiltration of mononuclear cells (lymphocytes, plasma cells and macrophages) in the lungs; bile duct proliferation and/or infiltration of mononuclear cells in the portal areas of the liver; infiltration of mononuclear cells in lamina propria and/or around blood vessels in the intestines; infiltration of mononuclear cells in the kidney; infiltration of mononuclear cells in plexus choroideus and/or perivascular cuffing of mononuclear cells in the meninges or brain parenchyma.

Lesions in lung, liver, intestine, kidney and brain were scored as: suspected (one small, focal infiltration of mononuclear cells); mild (few and small infiltrations); moderate (several small or few disseminated infiltrations); or massive (multifocal disseminated infiltrations). The spleen was evaluated for the following AMDV associated lesions: activation of lymphoid follicles with necrotic cells and macrophages; and reduced density (depletion) of lymphoid cells in lymphoid follicles and/or diffusely in the spleen. In the mesenteric lymph node, the following lesions were described: reduced density of lymphoid cells in lymphoid follicles; reduced density of lymphocytes in medulla and increased numbers of plasma cells in medulla and sinuses; increased numbers of macrophages, mainly in the medulla of the lymph node.

Due to the limited number of mink in each dilution group in the short term study, the histopathology of the mink euthanized at 2 weeks after AMDV inoculation (n = 4) is described collectively and likewise for the mink euthanized at 4 weeks (n = 8). In addition, the histopathological changes are described collectively for the infected sentinel sapphire mink (n = 4) without regard for the differences in duration of the infection since the exact time of infection is not known.

QIAamp Blood Mini kit (Qiagen, Hilden, Germany) was used to isolate total DNA from mink organs, blood samples stabilized with EDTA and swab samples in PBS. Total DNA from fecal samples was isolated with QIAamp DNA Stool Mini Kit (Qiagen, Hilden, Germany). All samples were tested by PCR as described by Jensen et al. [18]. Sera were tested by CIEP at Kopenhagen Diagnostic (Kopenhagen Fur, Copenhagen, Denmark, [24]).

## Results

### Short term experimental infection with AMDV

The 12 mink inoculated with four different dilutions (undiluted, 1:100, 1:1000 and 1:10,000) of the AMDV organ homogenate developed antibodies as detected by CIEP after 2–3 weeks. These antibodies persisted until the animals were euthanized (Table 1). Too few mink were inoculated with each of the four dilutions (n = 3) to draw any solid conclusions between the groups. However, the mink given the undiluted AMDV organ homogenate all (n = 3) had seroconverted 2 weeks after AMDV inoculation whereas only one had seroconverted after AMDV inoculation with a 1:10,000 dilution (Table 1). AMDV DNA was found in fecal samples in all mink from 2 to 4 weeks after inoculation (Table 1). AMDV DNA was found in oro-nasal swabs 3 weeks after AMDV inoculation of mink with 1:100 and 1:1000 AMDV organ homogenate dilutions, and in the two kits 4 weeks after inoculation of the mothers. No AMDV DNA was found in oro-nasal swabs from mink inoculated with the undiluted and 1:10,000 dilution of the AMDV organ homogenate (Table 1). Viremia results are not shown because different blood preparations from different experimental days were used for PCR (plasma, serum, buffy coat and full blood) which made the results incomparable. No significant clinical signs related to AMDV infection were observed (data not shown). At necropsy 2 weeks after AMDV inoculation all mink (n = 4) had pale or enlarged livers, this was only seen in two of eight mink 4 weeks after AMDV inoculation (Table 2). In addition, splenomegaly was seen in six of eight mink 4 weeks after AMDV inoculation (Table 2).

**Table 1:** Short term experimental infection with different dilutions of Aleutian mink disease virus (AMDV) organ homogenate

Dilution of AMDV organ	Antibody detected by CIEP		Virus excretion oro-nasal		Virus excretion fecal		
Homogenate	W2	W3 + 4^a^	W3	W4	W2	W3	W4
1:10,000	1/3	2/2	0/2	0/2	1/3	2/2	2/2
1:1000	2/3	2/2	1/2	2/2	2/3	2/2	2/2
1:100	2/3	2/2	1/2	1/2	1/3	2/2	2/2
Undiluted	3/3	2/2	0/2	0/2	2/3	0/2	1/2^b^
In total	8/12	8/8	2/8	3/8	6/12	6/8	7/8
Kits 3w old (n = 2)	na	na	na	2	na	na	na

Number of AMDV positive mink after AMDV inoculation out of total number of mink

*CIEP* counter current immunoelectrophoresis, *w* week, *na* not assessed

^a^Week (W) 3 and 4 were merged because the results were the same

^b^Rectal swab was positive

**Table 2:** Necropsy findings of Aleutian mink disease virus (AMDV) inoculated mink (n = 47, including sentinels)

Weeks after AMDV	Mink with gross pathological changes			
Inoculation	Fur^a^	Liver^b^	Spleen^c^	Kidney^d^
2 weeks	0	4/4	0/4	0/4
4 weeks	0	2/8	6/8	0/8
8 weeks	0	9/11	7/11	4/11
16 weeks	0	10/12	6/12	1/12
24 weeks	2/12	3/12	2/12	0/12

^a^Abnormal fur colour with sprinklers

^b^Abnormal livers were pale and some mink also had congestive hepatopathy

^c^Enlarged spleen

^d^Either pale cortex, enlarged or both

AMDV was found by PCR in all types of examined organs after euthanasia, though not with the same frequency (Table 3). Most consistently, AMDV was found in the spleen, mesenteric lymph node, kidney, liver and bone marrow, all mink euthanized after 4 weeks had virus in these organs (Table 3).

**Table 3:** Aleutian mink disease virus (AMDV) DNA detected with PCR in organs of mink

Organs	Number of mink with AMDV positive organs		
	2 weeks	4 weeks	4 weeks, kits 3w old^a^
Spleen	4/4	8/8	2/2
Mesenteric lymph node	3/4	8/8	2/2
Liver	4/4	8/8	2/2
Lung	2/4	6/8	2/2
Kidney	3/4	8/8	2/2
Intestine	3/4	7/8	2/2
Brain	2/4	6/8	na
Bone marrow	3/4	8/8	na

The groups of mink inoculated with different AMDV dilutions were merged in the table because the results of the small groups were similar

*na* not assessed

^a^Two mink kits were born the first week of the experiment. The organs of these two kits were tested with PCR for AMDV DNA 3 weeks later

A summary of the histopathology results of the short term study are presented in Table 4. Solid conclusions were difficult to obtain due to the small number of mink in these first weeks. Briefly, the majority of the mink had depletion of lymphoid cells in the spleen and mesenteric lymph nodes within the first 4 weeks after inoculation with AMDV. Activation of the lymphoid follicles in the spleen was another frequent finding at 4 weeks after AMDV inoculation (five of eight mink). Increased numbers of plasma cells and/or macrophages in the mesenteric lymph nodes were observed in about half of the AMDV inoculated mink. All mink (n = 12) had periportal infiltration of mononuclear cells in the liver, with a tendency of more pronounced lesions at 4 weeks, whereas lung- and kidney lesions were only seen at 4 weeks after inoculation. In the brain only mild lesions of non-suppurative perivascular cuffing and leptomeningitis was seen in five of eight mink at 4 weeks and one of four mink at 2 weeks. The cellular infiltration in lung, kidney, liver and brain was a mixture of plasma cells, lymphocytes and macrophages. Both mink kits had non-suppurative interstitial pneumonia without hyaline membranes, and one of them had massive periportal infiltration of mononuclear cells in the liver, whereas the other had bile duct proliferation.

**Table 4:** Scoring of histopathological changes in Aleutian mink disease virus (AMDV) inoculated mink

Tissue	Score/lesion	Sentinel	Post inoculation week				
			2	4	8	16	24
Lung	Suspect	–	–	–	–	–	–
	Mild	–	–	2	2/9	2/10	–
	Moderate	1	–	–	–	2/10	–
	Massive	1	–	2	2/9	–	–
	Total	2/4 (50 %)	0/4	4/8 (50 %)	4/9 (44 %)	4/10 (40 %)	0/10
Liver	Suspect	–	–	–	1/9	3/9	1/9
	Mild	–	2	2	2/9	4/9	3/9
	Moderate	3	2	2	5/9	1/9	3/9
	Massive	1	–	4	1/9	–	–
	Total	4/4 (100 %)	4/4 (100 %)	8/8 (100 %)	9/9 (100 %)	8/9 (89 %)	7/9 (78 %)
Intestine	Suspect	–	–	–	–	–	–
	Mild	–	–	–	–	–	1/10
	Moderate	1	–	–	3/7	–	–
	Massive	–	–	–	–	–	–
	Total	1/4 (25 %)	0/4	0/7	3/7 (43 %)	0/10	1/10 (10 %)
Kidney	Suspect	–	–	–	1/9	3/10	2/10
	Mild	1	–	2	2/9	1/10	2/10
	Moderate	–	–	2	2/9	1/10	2/10
	Massive	3	–	2	1/9	3/10	–
	Total	4/4 (100 %)	0/4	6/8 (75 %)	6/9 (67 %)	8/10 (80 %)	6/10 (60 %)
Brain	Suspect	–	–	–	1/9	–	–
	Mild	2	1	5	6/9	3/10	6/10
	Moderate	–	–	–	–	2/10	–
	Massive	–	–	–	–	–	–
	Total	2/4 (50 %)	1/4 (25 %)	5/8 (63 %)	7/9 (78 %)	5/10 (50 %)	6/10 (60 %)
Spleen	LF act^a^	–	–	5/8	2/9	4/10	–
	LF dep^b^	3/4	3/4	1/8	–	2/10	2/10
	Dif. dep^c^	2/4	–	1/8	1/9	2/10	3/10
	Stasis	1/4	2/4	2/8	2/9	2/10	1/10
	Total^d^	4/4 (100 %)	4/4 (100 %)	8/8 (100 %)	3/9 (33 %)	8/10 (80 %)	4/10 (40 %)
Mesenteric lymph node	LF dep	3/4	3/4	4/7	4/9	1/9	1/9
	Plasma cells^e^	2/4	3/4	3/7	3/9	3/9	7/9
	Macrop^f^	2/4	1/4	3/7	–	3/9	3/9
	Total^g^	4/4 (100 %)	3/4 (75 %)	7/7 (100 %)	6/9 (67 %)	5/10 (50 %)	7/9 (78 %)

No. of animals with lesion/no. of tissues examined

In lung, liver, intestine, kidney and brain the grade of the lesions/infiltrations of mononuclear cells were scored semi quantitatively as follows; Suspect: single small lesion. Mild, moderate and massive, respectively: small, moderate, massive lesion/infiltration of mononuclear cells

^a^Activation of lymphoid follicles (LF act) with presence of necrotic cells and macrophages

^b^Depletion of lymphoid cells in LF (LF dep)

^c^Diffuse depletion of lymphoid cells (Dif dep)

^d^Total number of mink with histological changes in the spleen

^e^Reduced density of lymphocytes and increased numbers of plasma cells in the sinuses (plasma cells)

^f^Increased numbers of macrophages, mainly in the medulla (macrop)

^g^Total number of mink with histopathological changes in the mesenteric lymph node

### Chronic experimental infection with AMDV

In total, 3894 mink days were scored, meaning every day each mink was observed in total of all mink in the experiment. Overall, at 92.7 % of the mink days the mink were clinically normal. During 7.3 % of the mink days, abnormal clinical scores were recorded, primarily within the first 8 weeks (September and October) of the experiment (data not shown because of unspecific findings). At the majority of mink days with abnormal scoring, the mink were generally in good condition (n = 233 mink days, 82 %), but either having soft or discolored feces (n = 142, 50.2 %) or decreased appetite (n = 46, 16.3 %). The majority of the mink with compromised general condition was in addition observed to have slightly decreased appetite (30 of 50 mink days, data not shown). No hematuria was observed.

Three mink (one wild type and two sapphire mink) were euthanized during the experiment due to reaching the clinical endpoints. About five weeks after AMDV inoculation one wild type female (from the group of mink 8 weeks in the experiment) had decreased appetite, was reluctant to move and had soft feces. Three days later, it had labored respiration and was euthanized. The necropsy findings were pale liver and enlarged pale congested kidneys. The results from this mink were included in the 8 weeks group. One sapphire female, developed an ulcer on the nose appearing around 8 weeks after the experiment started and was treated with antibiotics. Later it developed conjunctivitis and 1 week later was slow and had slightly decreased appetite, it deteriorated with anorexia and diarrhea and was euthanized. At necropsy, the liver was pale and hemolytic *Staphylococci* sp. was cultured. Another sapphire mink had slightly decreased appetite 16 weeks after the experiment started and developed nasal and ocular discharged and was euthanized few days later. At gross necropsy, the findings were gingivitis, enlarged congested spleen and the kidneys had pale cortex.

In general, few pathological findings were observed at necropsy (Table 2). The majority of the findings were pale liver and splenomegaly 8–16 weeks after AMDV inoculation, less frequently pale or enlarged kidneys were seen (Table 2). Two of the mink euthanized 24 weeks after AMDV inoculation had single “sprinklers”. Details of serology and virology results of the chronic experimental infection are described by Jensen et al. [24]. In short, the mink seroconverted 2–3 weeks after AMDV inoculation and remained seropositive throughout the 24 weeks of experiment. The majority of the mink had a transient viremia developing 1–3 weeks after AMDV inoculation and two mink had persistent viremia for 24 weeks. AMDV excretion was detected in feces and oro-nasal swabs at various and intermittent time intervals [24].

### Histopathology observed 8, 16 and 24 weeks after AMDV inoculation

Histopathological changes typical for AMDV infection was seen in all organs from mink in the long term study, a summary of these results are presented in Table 4 and Fig. 2. The majority of the mink had histopathological changes in the liver and kidney, and less consistently in the lung and intestine (Table 4). The involvement of the different organs and the extent of the lesions varied within and between the groups of animals. There was no statistical significant difference throughout the study period (early compared to late) in the number of histopathological findings in the lung, liver, kidney or brain (data not shown).

Two of the sentinel sapphire mink did not become viremic or developed antibodies and these two mink had no AMDV associated histopathological changes. Nevertheless, one of them developed slight depletion of lymphocytes and had increased numbers of plasma cells and macrophages in the mesenteric lymph node. Infiltration of mononuclear cells was seen in liver and kidney of the four viremic sentinel mink, and two of these had perivascular infiltrations in the lung. Other findings were perivascular cuffings (one mink) and mononuclear infiltrations in plexus choroideus (one mink) in the brain, and intestinal perivascular infiltrations of mononuclear cells (one mink).

The two wild type mink with persistent viremia throughout the 24 weeks of the study had histopathological changes similar to the other mink. They both had few and small infiltrations with monocular cells in the lungs and moderate in the liver. One of the mink had moderate multifocal infiltrations in the kidneys and some dilated tubuli and mild non-suppurative leptomeningitis. The other mink had mineral deposits in the renal tubuli and mild infiltrations with mononuclear cells in the intestines.

Two mink were euthanized during the infection experiment due to clinical disease, one wild type mink after 5 weeks and a sapphire mink after 18 weeks. These two mink had histopathological changes similar to the others: Moderate non-suppurative perivascular infiltrations of mononuclear cells in the lungs, and liver. Mild infiltrations in the intestines and massive infiltrations in the kidneys. In addition, the sapphire mink also had mild mononuclear cell infiltrations in the brain. No correlation between histopathology and AMDV DNA in the organs compared to the other mink could be established.

## Discussion

The AMDV infection induced in this study provide a model for chronic AMDV infection in mink with a 1:10,000 dilution of an organ homogenate with the currently circulating Danish AMDV strain, Saeby/DEN/799.1/05. Surprisingly, even with undiluted AMDV organ homogenate the mink did not become clinically affected within four weeks, indicating that the Saeby/DEN/799.1/05 is not dose dependent. In the short time study, irrespective of the inoculation dosage, the mink developed antibodies 2–4 weeks after AMDV inoculation, like in the long term study [24]. Similar, all mink in the short term study excreted AMDV viral DNA in feces as shown for the long term study [24]. AMDV DNA excretion in oro-nasal secretions was detected in the mink inoculated with AMDV diluted 1:1000 and 1:100, but not undiluted and diluted 1:10,000 supporting the previously observed inconsistency in excretion [24, 25]. AMDV DNA was found in all examined organs 2 and 4 weeks after AMDV inoculation, which is consistent with another study where AMDV DNA also was found in spleen, lymph nodes, bone marrow, liver, kidney, lung and intestines 10 days after inoculation with a Canadian AMDV field strain [19]. However, the number of mink in the short term experiment was low but the apparent lack of doses dependent response in infection confirms earlier studies [7, 20] and contradicts an older study of dose dependency in AMDV infection [25]. The lowest dilution of AMDV organ homogenate was chosen for the long term experiment because the lowest dilution was enough to induce chronic infection which was the aim of the study. Using a higher dilution of the AMDV organ homogenate potentially could induce an acute infection although the short term study did not indicate it. Given the ethical considerations of an animal experiment and economic expenses no such risk were taken and the lowest AMDV organ homogenate dilution was used. Unexpectedly, one mink delivered two kits which were kept for 3 weeks and both of these kits were subclinically infected with AMDV, i.e. the organs were PCR positive and they excreted AMDV DNA. The kits did not present symptoms in contrast to previous findings [10, 11] maybe because of their short life.

The clinical signs observed in this study were scarce with 93 % of all observations representing unaffected mink in good condition, probably due to the chronic subclinical course of the experimental infection. This was also observed in another experimental infection study [12] and under commercial farm conditions (unpublished observation, authors). Secondly, mink were euthanized within short time after showing the first symptoms for animal welfare reasons, so clinical AD was not observed in progression. The initial diffuse symptoms of decreased appetite corresponded to what Eklund et al. [25] observed. The three mink euthanized for welfare reasons were most likely suffering from AD, although hemolytic Staphylococcus was cultured in the liver from one of the mink. This relatively low disease rate is comparable to what has been observed in other chronic experimental AMDV infection of non-Aleutian genotype mink [5, 8, 12, 20]. Also in farms with chronic AMDV infection clinical signs and mortality in general is relatively limited [26] and the reason why commercial mink farms can produce despite AMDV infection. Interestingly, the majority of the clinical signs in the present study were observed in the first 2 months of the experiment, September and October. The relatively high prevalence of symptoms in these months can be related to stress after being moved to the experimental facility and change to winter fur (October). In commercial farms the antibiotic usage is commonly prescribed in these months which indicate this is a stressful period for the mink under production conditions, too [27].

Notably, no central nervous system (CNS) symptoms were observed despite the infiltrations of mononuclear cells in cerebrum, cerebellum, meninges and plexus choroideus. This is consistent with a study of Irish mink farms without neurologic disease, but non-suppurative meningoencephalitis [16], but contrary to an American field study of farmed mink with neurologic symptoms caused by non-suppurative meningoencephalitis [15]. The absence of CNS symptoms might be explained by the apparently less severe CNS lesions in the present study compared to the lesions described by Dyer et al. [15] which could be a time related issue.

The sentinel sapphire mink were included in the comparison of clinical signs and gross pathology after natural transmission of the virus knowing that they were not infected at the same time as the other mink. However, they showed the same symptoms and pathology as the experimentally inoculated mink. Clinical disease led to euthanasia of two of six sapphire mink and only one of 29 wild type mink presumably due to the sapphire genotype [7, 8]. Overall, the sapphire mink were too few for firm conclusions in itself, but they represented important results as sentinels in the experimental infection. Compared to previous studies it was expected that the sapphire mink would be more affected by AMDV than the wild type mink [7, 8, 28]. Not only the mink genotype but also virus strain of AMDV has been associated with different virulence [6–8]. The AMDV-Utah and AMDV-TR caused progressive disease in sapphire and pastel mink whereas AMDV-Pullman caused only progressive disease in 2/3 of the sapphire mink and not at all in the pastel mink [8]. Hadlow et al. found no consistent difference in clinical signs between the AMDV strains [7]. However, it is believed that many of the field strains of AMDV has a lower virulence [19, 20, 26] compared to the AMDV strains used in older studies [6].

The gross pathology observed in this study was similar regardless of how long time after AMDV inoculation the mink were euthanized with exception of: (1) the white sprinkle hairs that was observed only 24 weeks after AMDV inoculation, and (2) the kidney lesions were not observed until 2 months after AMDV inoculation. In general, few pathological changes are observed in chronic AMDV infected mink [14], which might represents the unspecific symptoms observed.

The AMDV associated histopathology seen in the present study corresponds to that seen by others [14–16, 28, 29]. No pathognomonic lesions exist for AD, but infiltration of mononuclear cells in several organs raises a strong suspicion of AD. The earliest histopathological sign of AMDV infection was seen in the liver at 2 weeks after inoculation, which has also been shown by others [28]. This might be related to the intraperitoneal route of inoculation, causing the virus to reach the liver as the first organ compared to for example the lung or the kidneys. This is further supported by the fact that no AMDV related lesions were seen in the lung or kidney of the mink 2 weeks in experiment, whereas these tissues developed AMDV related lesions later in the experimental infection. Furthermore, the liver was the organ that most often had lesions in all groups of mink, followed by the kidneys, brain, lung and intestines, respectively. This is in contrast to the prevalence of AMDV DNA in the organs, where the intestines were much more frequently positive compared to liver tissue at all time points [24] and indicates that the lesions are not caused by the viral infection itself, but the increased antibody production.

In the short term study the lesions were seen more frequent and pronounced in the mink euthanized 4 weeks after AMDV inoculation compared to the mink euthanized after 2 weeks, which reflects the time it takes to develop the lesions. The histopathological changes seemed to decrease from weeks 8 to 24 in the long term study with the exception of fur/skin affection, the same tendency was seen in regard to gross lesions. However, these differences were not statistically significant and the small sample size excludes firm conclusions. Histology is an important and sensitive tool in regard of suspicion and diagnostics of AD; however, final diagnosis must always be confirmed by PCR and/or antibody detection. Due to the variations in the progression of the lesions, histology cannot be used to conclude the time of infection.

## Conclusions

The currently circulating AMDV strain Saeby/DEN/799.1/05 cause few clinical signs with low mortality after experimental infection. The clinical signs observed were primarily decreased appetite and soft and/or discolored feces. The gross pathology was mainly associated with liver, spleen and kidney. Histopathological changes were seen in all organs but most frequently in liver, kidney and brain. No specific time of onset or pathognomonic lesions was observed. These findings are consistent with previous studies of AMDV and highlight the unpredictable disease pattern of AMDV and the necessity of supplementary and more sensitive tests like PCR and/or serology to confirm the diagnosis.

## References

[1] Knuuttila A, Uzcategui N, Kankkonen J, Vapalahti O, Kinnunen P (2009). Molecular epidemiology of Aleutian mink disease virus in Finland. Vet Microbiol.

[2] Sang Y, Ma J, Hou Z, Zhang Y (2012). Phylogenetic analysis of the VP2 gene of Aleutian mink disease parvoviruses isolated from 2009 to 2011 in China. Virus Genes.

[3] Farid AH, Zillig ML, Finley GG, Smith GC (2012). Prevalence of the Aleutian mink disease virus infection in Nova Scotia, Canada. Prev Vet Med.

[4] Christensen LS, Gram-Hansen L, Chriél M, Jensen TH (2011). Diversity and stability of Aleutian mink disease virus during bottleneck transitions resulting from eradication in domestic mink in Denmark. Vet Microbiol.

[5] Porter DD, Larsen AE, Porter HG (1980). Aleutian disease of mink. Adv Immunol.

[6] Alexandersen S (1990). Pathogenesis of disease caused by Aleutian mink disease parvovirus. APMIS Suppl.

[7] Hadlow WJ, Race RE, Kennedy RC (1983). Comparative pathogenicity of four strains of Aleutian disease virus for pastel and sapphire mink. Infect Immun.

[8] Oie KL, Durrant G, Wolfinbarger JB, Martin D, Costello F, Perryman S (1996). The relationship between capsid protein (VP2) sequence and pathogenicity of Aleutian mink disease parvovirus (ADV): a possible role for raccoons in the transmission of ADV infections. J Virol.

[9] Larsen S, Alexandersen S, Lund E, Have P, Hansen M (1984). Acute interstitial pneumonitis caused by Aleutian disease virus in mink kits. Acta Pathol Microbiol Immunol Scand [A].

[10] Alexandersen S (1986). Acute interstitial pneumonia in mink kits: experimental reproduction of the disease. Vet Pathol.

[11] Alexandersen S, Larsen S, Aasted B, Uttenthal A, Bloom ME, Hansen M (1994). Acute interstitial pneumonia in mink kits inoculated with defined isolates of Aleutian mink disease parvovirus. Vet Pathol.

[12] Alexandersen S, Bloom ME, Wolfinbarger J (1988). Evidence of restricted viral replication in adult mink infected with Aleutian disease of mink parvovirus. J Virol.

[13] Porter DD, Larsen AE, Porter HG (1973). The pathogenesis of Aleutian disease of mink. 3. Immune complex arteritis. Am J Pathol.

[14] Valdovska A, Pilmane M (2011). Histopathologic and immunohistochemical lesions in liver of mink infected with Aleutian disease virus. Pol J Vet Sci.

[15] Dyer NW, Ching B, Bloom ME (2000). Nonsuppurative meningoencephalitis associated with Aleutian mink disease parvovirus infection in ranch mink. J Vet Diagn Invest.

[16] Jahns H, Daly P, McElroy MC, Sammin DJ, Bassett HF, Callanan JJ (2010). Neuropathologic features of Aleutian disease in farmed mink in Ireland and molecular characterization of Aleutian mink disease virus detected in brain tissues. J Vet Diagn Invest.

[17] Farid AH, Ferns LE (2011). Aleutian mink disease virus infection may cause hair depigmentation. Scientifur.

[18] Jensen TH, Christensen LS, Chriel M, Uttenthal A, Hammer AS (2011). Implementation and validation of a sensitive PCR detection method in the eradication campaign against Aleutian mink disease virus. J Virol Methods.

[19] Farid AH, Hussain I, Arju I (2015). Detection of Aleutian mink disease virus DNA and antiviral antibodies in American mink (*Neovison vison*) 10 days postinoculation. J Vet Diagn Invest.

[20] Jackson MK, Ellis LC, Morrey JD, Li ZZ, Barnard DL (1996). Progression of Aleutian disease in natural and experimentally induced infections of mink. Am J Vet Res.

[21] An SH, Ingram DG (1978). Transmission of Aleutian disease from mink with inapparent infections. Am J Vet Res.

[22] Johnson MI, Henson JB, Gorham JR (1975). The influence of genotype on the development of glomerular lesions in mink with Aleutian disease virus. Am J Pathol.

[23] Mori S, Nose M, Miyazawa M, Kyogoku M, Wolfinbarger JB, Bloom ME (1994). Interstitial nephritis in Aleutian mink disease. Possible role of cell-mediated immunity against virus-infected tubular epithelial cells. Am J Pathol.

[24] Jensen TH, Hammer AS, Chriél M (2014). Monitoring chronic infection with a field strain of Aleutian mink disease virus. Vet Microbiol.

[25] Eklund CM, Hadlow WJ, Kennedy RC, Boyle CC, Jackson TA (1968). Aleutian disease of mink: properties of the etiologic agent and the host responses. J Infect Dis.

[26] Jackson MK, Winslow SG, Dockery LD, Jones JK, Sisson DV (1996). Investigation of an outbreak of Aleutian disease on a commercial mink ranch. Am J Vet Res.

[27] Chriél M, Harslund J, Jensen TH, Jakobsen E, Hammer AS, Lassen-Nielsen AM (2012). Antimicrobials in mink-consumption and resistance patterns. Proceedings of the 10th International Scientific Congress in fur animal production. Scientifur.

[28] Henson JB, Gorham JR, McGuire TC, Crawford TB (1976). Pathology and pathogenesis of Aleutian disease. Front Biol.

[29] Bloom ME, Kanno H, Mori S, Wolfinbarger JB (1994). Aleutian mink disease: puzzles and paradigms. Infect Agents Dis.

